# A Review: Inflammatory Process in Alzheimer's Disease, Role of Cytokines

**DOI:** 10.1100/2012/756357

**Published:** 2012-04-01

**Authors:** Jose Miguel Rubio-Perez, Juana Maria Morillas-Ruiz

**Affiliations:** Department of Food and Nutrition Technology, St. Anthony Catholic University, Campus de Los Jerónimos, s/n Guadalupe, 30107 Murcia, Spain

## Abstract

Alzheimer's disease (AD) is the most common neurodegenerative disorder to date. Neuropathological hallmarks are **β**-amyloid (A**β**) plaques and neurofibrillary tangles, but the inflammatory process has a fundamental role in the pathogenesis of AD. Inflammatory components related to AD neuroinflammation include brain cells such as microglia and astrocytes, the complement system, as well as cytokines and chemokines. Cytokines play a key role in inflammatory and anti-inflammatory processes in AD. An important factor in the onset of inflammatory process is the overexpression of interleukin (IL)-1, which produces many reactions in a vicious circle that cause dysfunction and neuronal death. Other important cytokines in neuroinflammation are IL-6 and tumor necrosis factor (TNF)-**α**. By contrast, other cytokines such as IL-1 receptor antagonist (IL-1ra), IL-4, IL-10, and transforming growth factor (TGF)-**β** can suppress both proinflammatory cytokine production and their action, subsequently protecting the brain. It has been observed in epidemiological studies that treatment with nonsteroidal anti-inflammatory drugs (NSAIDs) decreases the risk for developing AD. Unfortunately, clinical trials of NSAIDs in AD patients have not been very fruitful. Proinflammatory responses may be countered through polyphenols. Supplementation of these natural compounds may provide a new therapeutic line of approach to this brain disorder.

## 1. Introduction

Originally described by Alois Alzheimer in 1907 [1], AD is now the most common cause of dementia in the elderly. AD affects more than 4 million people in the United States [2]. It is estimated that 27 million people are affected worldwide [3]. As population life expectancy increases, the number of affected individuals is expected to triple by 2050 [2].

AD is a progressive brain disorder affecting regions of the brain that control memory and cognitive functions, gradually destroying a person's memory and ability to learn, to reason, to communicate, and to carry out daily activities. 

The two major neuropathologic hallmarks of AD are extracellular A*β* plaques and intracellular neurofibrillary tangles. The production of A*β*, a seminal event in AD [4], is a result of the cleavage of the amyloid precursor protein (APP), volume of which is high in AD. APP has important developmental functions in cell differentiation and possibly in the establishment of synapses [5, 6], but the function of APP in the adult brain is less clear. What we do know, however, is that it is expressed by neurons in response to cell injury. APP is, for example, a marker for axonal damage after head injury [7, 8]. APP expression is markedly increased in the affected areas of the brain in temporal lobe epilepsy [9]. Neurofibrillary tangles are composed of the tau ( *τ* ) protein. In healthy neurons, *τ* is an integral component of microtubules, which are the internal support structures that transport nutrients, vesicles, mitochondria, and chromosomes from the cell body to the ends of the axon and backwards. In AD, however, *τ* becomes hyperphosphorylated. This phosphorylation allows *τ* to bind together and form tangled threads [10].

Gliosis is also seen in AD; activated astrocytes and microglia are characteristically found in abundance near neurons and plaques. Once activated, astrocytes and microglia produce several proinflammatory signal molecules, including cytokines, growth factors, complement molecules, chemokines, and cell adhesion molecules [11–15]. This activation is thought to result from the glial reaction to the events related to the ongoing deposition of A*β* [16–18].

## 2. Inflammatory Process in Alzheimer's Disease

Inflammation is a response to eliminate both the initial cause of cell injury as well as the necrotic cells and tissues resulting from the original insult. If tissue health is not restored, inflammation becomes a chronic condition that continuously erodes the surrounding tissues. In this type of inflammation, tissue injury and healing proceed simultaneously. The lateral damage normally caused tends to accumulate slowly, sometimes even asymptomatically during years. This can lead to severe tissue deterioration [19].

Brain inflammation is a pathological hallmark of AD. However, the characteristic inflammatory features such as swelling, heat, and pain are not present in the brain, and therefore we refer here to chronic instead of acute inflammation [14]. A characteristic feature of chronic inflamed tissues is the presence of an increased number of monocytes, as well as monocyte-derived tissue macrophages, that is, microglia cells in the central nervous system (CNS) [14, 19]. Inflammation clearly occurs in pathologically vulnerable regions of the AD brain, with increased expression of acute phase proteins and proinflammatory cytokines which are hardly evident in the normal brain [20–23]. Microglia, astrocytes, and neurons are responsible for the inflammatory reaction.

Activated cells strongly produce inflammatory mediators such as proinflammatory cytokines, chemokines, macrophage inflammatory proteins, monocyte chemo-attractant proteins, prostaglandins, leukotrienes, thromboxanes, coagulation factors, reactive oxygen species (and other radicals), nitric oxide, complement factors, proteases, protease inhibitors, pentraxins, and C-reactive protein [13, 14, 18, 24, 25].

The hypothesis is that the intractable nature of the A*β* plaques and tangles stimulates a chronic inflammatory reaction to clear this debris [25]. These plaques contain dystrophic neurites, activated microglia, and reactive astrocytes [14, 15, 26]. Aggregated amyloid fibrils and inflammatory mediators secreted by microglial and astrocytic cells contribute to neuronal dystrophy [27, 28]. Chronically activated glia can, furthermore, kill adjacent neurons by releasing highly toxic products such as reactive oxygen intermediates, nitric oxide (NO), proteolytic enzymes, complementary factors, or excitatory amino acids [29]. Inflammatory mediators and a number of stress conditions, in turn, enhance APP production and the amyloidogenic processing of APP to induce amyloid-*β*-42 (A*β*-42) peptide production. These circumstances also inhibit the formation of soluble APP fraction that has a neuronal protective effect [30–35]. On the other hand, A*β* induces the expression of proinflammatory cytokines in glia cells in a vicious cycle [18, 36], the activation of the complement cascade [37–39], and the induction of inflammatory enzyme systems such as the inducible nitric oxide synthase (iNOS) and the cyclooxygenase enzyme (COX)-2. Several lines of evidence suggest that all of these factors can contribute to neuronal dysfunction and cell death, either alone or in concert [40–42].

### 2.1. Microglia

Microglia constitute around 10% of the cells in the nervous system. They represent the first line of defense against invading pathogens or other types of brain tissue injury. Under pathological situations, such as neurodegenerative disease, stroke, traumatic injury, and tumor invasion, these cells become activated, migrate, and surround damaged or dead cells, and subsequently clear cellular debris from the area. This action is similar to the one performed by phagocytic active macrophages of the peripheral immune system [43].

The current evidence points compellingly towards a central role for inflammation in AD. This inflammation is mediated by proinflammatory cytokines and would create a chronic and self-sustaining inflammatory interaction between activated microglia and astrocytes, stressed neurons, and A*β* plaques.

Microglia have been suggested to be preferentially associated with certain amyloid plaque types [44]. Amyloid peptides and their precursor protein APP are potent glial activators [45, 46]. Disruption of the APP gene and its proteolytic products delay and decrease microglial activation [47]. This activation is directly dependent on the amyloid load. Treatment with *β*-sheet breakers peptide results in reduced brain inflammation [48].

A*β* is able to stimulate a nuclear factor-kappaB- (NF*κ*B-) dependent pathway that is required for cytokine production [49]. The subsequent activation of extracellular signal-regulated kinase (ERK) and mitogen-activated protein kinase (MAPK) pathways by A*β* binding to the microglial cell surface induces proinflammatory gene expression and leads to the production of cytokines and chemokines [50].

In some situations, the role of microglia has been found to be beneficial, since activated microglia can reduce A*β* accumulation by increasing its phagocytosis, clearance, and degradation [51, 52]. Microglia can also secrete a number of soluble factors, such as the glia-derived neurotrophic factor (GDNF), which are potentially beneficial to the survival of neurons [53]. It was proposed, therefore, that microglial activation by active immunization might be a valid mechanism for clearance of senile plaques [54]. However, because a human trial of A*β* immunization led in some patients to meningoencephalitis, this treatment has been discontinued [55]. It was recently found that nasal vaccination in mice was able to decrease A*β*. The extent of this reduction correlated with microglial activation, suggesting that this may be a promising approach for human A*β* immunization [56].

### 2.2. Astrocytes

Astrocytes are known to be important for A*β* clearance and degradation, for providing trophic support to neurons, and for forming a protective barrier between A*β* deposits and neurons [57]. The presence of large numbers of astrocytes associated with A*β* deposits in AD suggests that these lesions generate chemotactic molecules that mediate astrocyte recruitment.

Under certain conditions related to chronic stress, however, the role of astrocytes may not be beneficial. A report suggests that astrocytes could also be a source for A*β*, because they overexpress *β*-secretase of APP (BACE1) in response to chronic stress [57]. In vitro and in vivo experiments suggest though that inflammatory active astrocytes do not generate significant amounts of these molecules.

### 2.3. Complement System

The complement system represents a complex and tightly regulated attack system designed to destroy invaders and to assist in the phagocytosis of waste materials. The components of this system carry out four major functions: recognition, opsonization, inflammatory stimulation, and direct killing through the membrane attack complex [58]. Complement proteins interact with cell surface receptors to promote a local inflammatory response that contributes to the protection and healing of the host. Complement activation causes inflammation and cell damage, yet it is essential for eliminating cell debris and potentially toxic protein aggregates [59].

The complement system consists of some 30 fluid-phase and cell-membrane-associated proteins that can be activated by different routes. The classical pathway (involving C1q, C1r, C1s, C4, C2, and C3 components) is activated primarily by the interaction of C1q with immune complexes (antibody antigen), but activation can also be achieved after interaction of C1q with nonimmune molecules such as DNA, RNA, C-reactive protein, serum amyloid P, bacterial lipopolysaccharides, and some fungal and virus membranes. The initiation of the alternative pathway (involving C3, factor B, factor D, and properdin) does not require the presence of immune complexes and leads to the deposition of C3 fragments on target cells. The molecular network of classical and alternative complement cascades with pattern recognition, proteolytic activation, functions of fragments in phagocytosis, and stimulation of the host immune defense has been reviewed in detail elsewhere [60–62].

Many complement proteins and receptors can be synthesized locally in the brain [63–66]. The complement system activation has been observed in brain in different inflammatory and degenerative diseases, for example, AD, multiple sclerosis, and stroke [59, 65, 67]. Surprisingly, the most potent complement defense in human brain seems to be located on the astrocytes which can express all the components of classical and alternative pathways, such as C1–C9, regulatory factors B, D, H, I, and several complement receptors, for example, C1qR, C3aR and C5aR [63, 65]. Microglial cells exhibit a more narrow set of complement proteins, for example, C1q, C3 and receptors C1qR, CR3, and C5aR, which support the phagocytic uptake of targeted structures. Interestingly, neurons also express several regulatory proteins, such as factors H and S, as well as receptors C1qR, C3aR and C5aR [65, 66, 68, 69].

Several research articles have reported that the complement system of brain is activated in AD [59, 67, 70, 71]; furthermore, this system seems to be activated at a very early stage of the disease. A*β* peptides can activate the complement cascade without the presence of antibody. They can additionally produce complement components [72]. C1q protein is mainly localized in neurons, along with neuritic plaques, both in the frontal cortex and in the hippocampus [73]. Interestingly, C1q protein is present only in thioflavin-positive amyloid plaques containing the *β*-sheet conformation [73] showing that C1q may affect the amyloid aggregation process.

Moreover, there is an extensive literature demonstrating that the complement system also has a neuroprotective role in neuroinflammation [59, 60, 71, 74]. For example, inhibition of the complement system could clearly increase amyloid plaque formation and the neurodegeneration occurring in transgenic AD mice [75]. Complement C3 knockout also aggravated the neuropathology in AD mice [76]. The activity of C1q protein in the clearance of apoptotic cells and A*β* aggregates into glial cells may well be the main cause for neuroprotection.

### 2.4. Chemokines

Recent experiments have focused on understanding the role of chemokines and their receptors for AD neuroinflammation.

The chemokine family consists of over 50 different molecules that confer chemotaxis, tissue extravasation, and modulation of leukocyte function during inflammation [77, 78]. The importance of chemokine generation in AD brain is underscored by the fact that these molecules may be strong regulators of microglial migration and recruitment of astrocytes to the area of neuroinflammation. They are therefore responsible for the extent of local inflammation.

While it has been reported that chemokines exert physiological action in the healthy brain [79], the majority of studies have focused on the expression pattern of chemokines and their respective receptors in neurological diseases such as multiple sclerosis, traumatic brain injury, and stroke. All of these disorders share the disruption of the blood-brain barrier as an important pathogenetic event subsequently allowing peripheral leukocytes to infiltrate the lesion site [80]. In contrast, no convincing evidence exists for blood-brain barrier disruption or significant leukocyte infiltration in the AD brain.

However, several chemokines and chemokine receptors have been found to be upregulated in the AD brain [81]. Chemokines may play an important role for recruiting microglia and astroglia to the site of A*β* deposition. A*β*-stimulated human monocytes generate chemokines such as IL-8, monocyte chemoattractant protein- (MCP)-1, macrophage inflammatory protein- (MIP)-1*α* and MIP-1*β* in vitro, and microglia cultured from rapid autopsies of AD and nondemented patients reveal an increased expression of IL-8, MCP-1, and MIP-1*α* after experimental exposure to A*β*. Supporting the hypothesis that astrocytes actively contribute to the inflammatory disease component, MIP-1*α* has been detected in reactive astrocytes nearby A*β* plaques.

### 2.5. Neurons

While neurons were traditionally believed to be passive bystanders in neuroinflammation, more recent evidence suggests that neurons can generate inflammatory molecules. Thus, neurons can serve as source of complement, COX-2-derived prostanoids [82–84], several cytokines [85–93], and macrophage colony-stimulating factor (MCSF) [94].

Although COX-2 expression is driven by physiological synaptic activity [93] and therefore may be regarded as physiologically expressed protein in a subclass of neurons, inflammation induced by the generation of prostanoids may well contribute to neuronal destruction. As a further factor, expression of the inflammatory-induced enzyme iNOS has been described in degenerating neurons in AD brains [95–97]. Compelling evidence exists also for iNOS-related long-term NO release and NO-dependent peroxynitrite formation [98]. Glial- and neuronal-derived NO and peroxynitrite have been demonstrated to cause neuronal dysfunction and cell death in vitro and in vivo [99, 100].

### 2.6. Cytokines

Cytokines are small and nonstructural proteins with molecular weights ranging from 8,000 to 40,000 Da. Originally called lymphokines and monokines to indicate their cellular sources, it soon became clear that the term “cytokine” was the best description, since nearly all nucleated cells are capable of synthesizing these proteins and, in turn, they are also capable of responding to these molecules. There is no amino acid sequence motif or three-dimensional structure that links cytokines. Their biological activities allow us in turn to group them into different classes.

Cytokines are secreted by a variety of immune cells (e.g., T-lymphocytes, macrophages, natural killer cells) and nonimmune cells (e.g., Schwann cells, fibroblasts). The biological effects induced by cytokines include the stimulation or inhibition of cell proliferation, cytotoxicity/apoptosis, antiviral activity, cell growth and differentiation, inflammatory responses, and upregulation of expression of surface membrane proteins. The main function of cytokines is the regulation of T-cell differentiation from undifferentiated cells to T-helper 1 and 2, regulatory T cells, and T-helper 17 cells [101]. These regulatory proteins include ILs, interferons (IFNs), colony stimulating factors (CSFs), TNFs, and certain growth factors (GFs) [102, 103].

Many of these cytokines have already been shown to be produced by neurons or glia and there are a number of reports indicating changes in their levels in AD brain, blood, and cerebrospinal fluid (CF). Levels of IL-1*α*, IL-1*β*, IL-6, TNF-*α*, granulocyte-macrophage colony-stimulating factor (GMSF), IFN-*α*, the type B of IL-8 receptor (IL-8RB), and the receptor for CSF-1 are reportedly increased in AD brain tissue [104, 105].

A number of interactions between cytokines and components of the AD senile plaques have been reported suggesting that a vicious circle might be generated [105]. Thus, the A*β* protein of the plaques is said to potentiate the secretion of IL-6 and IL-8 by IL-1*β*-activated astrocytoma cells, of IL-6 and TNF-*α* by lipopolysaccharide- (LPS-) stimulated astrocytes [106], and of IL-8 by monocytes. Cytokines can also stimulate secretion of a number of the other proteins found in senile plaques [105]. Moreover, synergistic effects may also occur between cytokines and A*β*. For example, IFN-*γ* is said to synergize with A*β* to cause the release of TNF-*α* and reactive nitrogen species that are toxic to neurons, and IL-1 is reported to increase the toxicity of A*β* in PC12 cells.

Some cytokines clearly promote inflammation and are called proinflammatory cytokines, whereas other cytokines suppress the activity of proinflammatory cytokines and are called anti-inflammatory cytokines. For example, IL-4, IL-10, and IL-13 are potent activators of B lymphocytes; however, IL-4, IL-10, and IL-13 are also potent anti-inflammatory agents. These are anti-inflammatory cytokines by virtue of their ability to suppress genes for proinflammatory cytokines such as IL-1, TNF, and the chemokines. IFN-*γ* is another example of the pleiotropic nature of cytokines. IFN-*γ* possesses antiviral activity, in the same way as IFN-*α* and IFN-*β*. IFN-*γ* is also an activator of the pathway affecting cytotoxic T cells; however, IFN-*γ* is considered a proinflammatory cytokine because it increases TNF activity and induces NO. The concept that some cytokines function would primarily be to induce inflammation while other cytokines primary function would be to suppress inflammation is fundamental to cytokine biology and also to clinical medicine.

The concept is based on the genes coding for the synthesis of small mediator molecules that are upregulated during inflammation. Therefore, a “balance” between the effects of proinflammatory and anti-inflammatory cytokines is thought to determine the outcome of disease, whether in the short term or long term. In fact, data from some studies suggest that susceptibility to disease is genetically determined by the balance or expression of either proinflammatory or anti-inflammatory cytokines. It should be considered though that some gene linkage studies are often difficult to interpret.

#### 2.6.1. Major Proinflammatory Cytokines

The cytokine class of inflammatory mediators is secreted by microglia and astrocytes surrounding A*β* neuritic plaques. Their production is increased in inflammatory states and they function by regulating the intensity and duration of the immune response [13]. The IL-1 family of cytokines includes two agonist proteins, IL-1*α* and IL-1*β*, which trigger cell activation upon binding with specific membrane receptors. Also included is IL-1ra, which is a glycosylated secretory protein of 23 kDa that counteracts the action of IL-1 [107].

IL-1 is an important initiator of the immune response, playing a key role in the onset and development of a complex hormonal and cellular inflammatory cascade. Elevated IL-1*β* has been detected in the CF and brain parenchyma within the early hours after brain injury in both humans and rodents [108, 109]. Nonetheless, IL-1 has been documented to play a role in neuronal degeneration. In astrocytes, IL-1 induces IL-6 production, stimulates iNOS activity [110], and induces the production of MCSF. In addition, IL-1 enhances neuronal acetylcholinesterase activity, microglial activation and additional IL-1 production, astrocyte activation, and expression of the beta-subunit of S100 protein (S100*β*) by astrocytes, thereby establishing a self-propagating cycle [18, 111].

IL-6 is a multifunctional cytokine that plays an important role in host defense [112], with major regulatory effects upon the inflammatory response [113]. IL-6 belongs to the neuropoietin family of cytokines [114], and it has both direct and indirect neurotrophic effects on neurons [115]. IL-6 promotes astrogliosis [116], activates microglia [117], and stimulates the production of acute phase proteins [118].

TNF-*α* plays a central role in initiating and regulating the cytokine cascade during an inflammatory response. It is produced as a membrane-bound precursor molecule of 26 kDa that is cleaved by the TNF-*α* converting enzyme to produce a 17 kDa active cytokine [119]. The levels of TNF-*α* expression in the healthy brain are low, making it difficult to determine its precise role under physiological conditions. In inflammatory or disease states, TNF-*α* along with several other proinflammatory mediators and neurotoxic substances are predominantly produced by activated microglia. Neuronal production of TNF-*α* has been demonstrated [86], although brain-derived TNF-*α* is mostly synthesized by glial cells in response to pathological stimuli. Glial cells secrete both TNF-*α* and IL-1, which in turn, activate these cells in an autocrine manner to induce further cytokine production and astrogliosis. TNF-*α*, on the other hand, has been reported to have neuroprotective properties [14] in the AD brain.

In addition to the general role of cytokines, AD-specific interactions of certain cytokines and chemokines with A*β* may be pathophysiologically relevant. For example, IL-1 can regulate APP processing and A*β* production in vitro [120]. In turn, fibrillar A*β* has been reported to increase neurotoxic secretory products, proinflammatory cytokines, and reactive oxygen species [121–123]. Cultured rat cortical glia exhibit elevated IL-6 mRNA after exposure to the carboxy-terminal 105 amino acids of APP [124]. In the same situation, IL-1, IL-6, TNF-*α*, MIP-1*α* and MCP-1 increase in a dose-dependent manner after cultured microglia are incubated with A*β*. The production of interleukins, other cytokines, and chemokines may also lead to microglial activation, astrogliosis, and further secretion of proinflammatory molecules and amyloid, thus perpetuating the cascade [50].

#### 2.6.2. Major Anti-Inflammatory Cytokines

A second general category of cytokine action is manifested by anti-inflammatory cytokines such as IL-1ra, IL-4, IL-10, and TGF-*β*. These inhibitory cytokines can suppress proinflammatory cytokine production and action, an effect that is critical to the concept of balance among pro- and anti-inflammatory cytokines. The clinical consequence of a CNS disregulation in this balance (high levels of proinflammatory cytokines, low levels or activity of anti-inflammatory cytokines) can lead to cytokine production and synergistic cytokine actions and can induce an amplification cycle of cellular activation and cytotoxicity [125]. Thus, both cytokine-cytokine interactions and cytokine interactions with existing AD pathology may play critical roles in AD neuroinflammation.

IL-1ra is a 152-amino-acid protein that functions as a specific inhibitor of the two other functional members of the IL-1 family, IL-1*α* and IL-1*β* [126, 127]. IL-1ra is produced by monocytes and macrophages and is released into the systemic circulation, blocking the action of IL-1*α* and IL-1*β* functional ligands by competitive inhibition at the IL-1 receptor level. IL-1ra binds with equal or greater affinity than does IL-1*α* and IL-1*β* to the type 1 (80 kDa) membrane-bound IL-1 receptor. In contrast, IL-1ra does not bind with high affinity to the type II (68 kDa) IL-1 receptor [128, 129].

The biological actions of IL-1*β* are regulated in vivo by IL-1ra [130]. This action is performed by preventing the binding of IL-1*β* to IL-1 type I receptor (IL-1RI) [131]. In vitro, IL-1ra suppresses IL-1*β*-induced TNF-*α* production and iNOS expression in astrocytes [132]. IL-1ra also protects against IL-1*β* neurotoxicity [133]. Furthermore, in vivo IL-1ra attenuates ischaemic and excitotoxic neuronal damage [134].

IL-4 is a 20-kDa glycoprotein produced by mature Th2 cells and cells from the mast cell or basophil lineage, which is able to influence Th-cell differentiation. IL-4 drives Th2 responses, mediates the recruitment and activation of mast cells, and stimulates the production of IgE antibodies via the differentiation of B cells into IgE-secreting cells [135, 136]. Also, IL-4 has marked inhibitory effects on the expression and release of the proinflammatory cytokines, it is able to block or suppress the monocyte-derived cytokines, including IL-1, TNF-*α*, IL-6, IL-8, and MIP-1*α* [135, 136], and it stimulates the synthesis of IL-1ra [137]. Other mechanism by which IL-4 exerts its neuroprotective effect might be related to the inhibition of IFN-*γ* and the consequent decrease in the concentration of TNF-*α* and NO [138].

IL-10 is one of the main anti-inflammatory cytokines. IL-10 mRNA is detectable in the frontal and parietal lobe of the normal brain [139] and has been suggested to play an important role in neuronal homeostasis and cell survival [140]. IL-10 mediates on cells by interacting with specific cell surface receptors (IL-10Rs), present on all the major glial cell populations in the brain [140], and it limits inflammation by reducing the synthesis of proinflammatory cytokines such as IL-1 and TNF-*α*, by suppressing cytokine receptor expression and by inhibiting receptor activation in the brain. A*β* does not seem to stimulate IL-10 production by glial cells in vitro [141], but preexposure of glial cells to IL-10 inhibits A*β*- or LPS-induced production of proinflammatory cytokines [139], suggesting that IL-10 receptors are present in cultured glial cells [142]. IL-10 inhibits monocyte/macrophage-derived TNF-*α*, IL-1, IL-6, IL-8, IL-12, GMSF, MIP-1*α*, and MIP-2*α* [143–145]. In addition to these activities, IL-10 attenuates surface expression of TNF receptors and promotes the shedding of TNF receptors into the systemic circulation [146, 147].

TGF-*β* is synthesized as an inactive precursor and requires activation before exerting its effect [148]. The active molecule is a 25-kDa homodimer of two 12.5-kDa disulfide-linked monomers and it belongs to a superfamily of >20 distinct dimeric proteins that share a similar structure [149]. TGF-*β* is an important regulator of cell proliferation, differentiation, and formulation of the extracellular matrix [150]. TGF-*β* is capable of converting an active site of inflammation into one dominated by reparations [150]. In addition, TGF-*β* suppresses the proliferation and differentiation of T cells and B cells and limits IL-2, IFN-*γ*, and TNF production.

All three known mammalian isoforms of TGF-*β*, that is, TGF-*β*1, 2, and 3, are expressed in the CNS and have been implicated in the pathogenesis of AD. TGF-*β* has been shown to modulate a wide range of processes that are implicated in AD, including brain injury response and astrocytosis, brain inflammatory response and microglial activation, extracellular matrix production, accumulation and regional distribution of amyloid, regulation of known or potential AD risk factors (e.g., APP, COX-2), and inhibition of cell death. For example, AD TGF-*β*1 has been detected in plaques [151], and higher TGF-*β*1 levels were found in cerebrospinal fluid [152] and serum [153] of AD cases than in nondemented controls. Immunostaining for TGF-*β*2 was observed in reactive astrocytes, ramified microglia, and a portion of tangle-bearing neurons in AD cases [154]. Finally, it should be noted that immunoreactivities for TGF-*β*1 and 2 receptors were higher in reactive glia in AD cases than in the nondemented controls [155].

### 2.7. Growth Factors

Growth factors are proteins, which support the survival of cells of the central and peripheral nervous system. Growth factors play a role in the development of the brain, they stimulate axonal growth and regulate the growth of different kinds of cells in the brain and periphery. In many cases, the same growth factor and corresponding receptor signalling system may thus serve a number of different functions in the body.

Nerve growth factor (NGF) is the most potent growth factor able to counteract cell death of cholinergic neurons in vitro and in vivo [156]. Increased NGF has been found in the CF of AD patients [157–159]. In spite of the fact that NGF disfunction has been suggested in the development of AD, NGF knockout mice have not shown clear cognitive deficits. NGF has been considered, nevertheless, as a candidate for treating AD purified NGF, in fact, it was infused in some AD patients [160]. NGF is upregulated in brains [161] and CSF [158] of AD patients, while the high-affinity NGF receptor trkA is downregulated [162]. Interestingly, the increase of NGF was specific for AD compared to healthy controls and was dependent on the extent of neurodegeneration as expressed by the phospho-tau181/A*β*-42 ratio [157]. Although NGF data alone did not reveal a significant difference, the comparison of NGF in AD patients having a phospho-tau181/A*β*-42 ratio >10 with healthy control subjects (ratio <6) revealed a significant difference [157]. This might suggest that NGF accumulates in neurodegeneration only at a certain stage of the disease.

Furthermore, vascular endothelial growth factor (VEGF) is an important growth factor, which regulates angiogenesis in the nervous system, and it is increased [163, 164] in AD, resulting in enhanced microvascular density when developing the disease. The disregulation of other growth factors may also contribute to AD. For example, platelet-derived growth factor (PDGF), which is mitogenic for cells of mesenchymal origin, has been found to upregulate APP in the hippocampus by inducing secretases [165–167]. Insulin-like growth factor-I (IGF-I) regulates A*β* levels and displays protective effects against A*β* toxicity [168, 169]. Finally, members of the TGF-*β* family interact with A*β*, contributing to its toxicity or constituting a risk for cerebral A*β* angiopathy [170, 171].

## 3. Anti-Inflammatory Therapy and Alzheimer's Disease

Based on the compelling evidence that inflammatory processes are involved in the pathogenesis of AD, research has looked into the use of anti-inflammatory drugs as a treatment option for patients with AD. Drugs such as the NSAIDs and glucocorticoid steroids have been studied to determine if they offer any benefits to AD patients.

### 3.1. NSAIDs

The NSAIDs are a family of drugs that include the salicylate, propionic acid, acetic acid, fenamate, oxicam, and the COX-2 inhibitor classes. They have analgesic, antipyretic, and anti-inflammatory properties by inhibiting the COX enzyme that catalyses the initial step in the conversion of arachidonic acid to several eicosanoids including thromboxanes, leukotrienes, and prostaglandins. Eicosanoids play major regulatory roles in cell functions including immune and inflammatory functions.

The COX enzyme is known to exist as two isoenzymes, COX-1 and COX-2, both of which occur in the brain but whose functions are not well understood. COX-1 is responsible for homeostatic production of prostanoids. COX-2 is inducible and its expression can be modified depending on the stimuli but may also have a role in the development of homeostasis [172]. With the exception of COX-2 inhibitors, all classes of NSAIDs inhibit both COX-1 and COX-2 enzymes. COX-2 inhibitors, as their name implies, selectively inhibit the COX-2 enzyme.

Epidemiological evidence indicates that NSAIDs may lower the risk of developing AD [173–176]. Since patients with rheumatoid arthritis and osteoarthritis are typically treated and are exposed to NSAIDs for a long period of time, epidemiological studies have looked into the association of these diseases and AD. Many of those studies showed an inverse relationship between having arthritis (and being treated with NSAIDs) and AD [177]. A prospective population-based study has also shown a significant reduction in the risk of AD in subjects who had taken NSAIDs for a cumulative period of 24 months or more [178]. Postmortem studies have also shown the ability of NSAIDs to reduce the inflammation that is consistently seen in AD brain tissue [179]. A possible mode of action for the effectiveness of NSAIDs is by the blockage of COX-2 in the brain. It has been shown that COX-2 mRNA is considerably upregulated in affected areas of AD brain [180, 181], with COX-2 immunoreactivity located mainly in pyramidal neurons in the cerebral cortex and the hippocampal formation [182], suggesting the involvement of COX-2 in AD.

The NSAIDs have been shown to directly affect the production of A*β* through several mechanisms. For example, ibuprofen, indomethacin, and sulindac sulphide were shown to decrease the A*β*-42 peptide by up to 80% in cultured cells (effect not observed with naproxen, celecoxib, or aspirin) [183]. Since not all NSAIDs had this effect, it would seem that this effect occurs through a process that is independent of their anti-inflammatory COX activity. Treatment of mice overexpressing APP with ibuprofen resulted in a reduction of the amyloid plaque load in the cortex along with a reduction of microglial activation in the mice [184]. A study analyzing the ability of common NSAIDs and the enantiomers of flurbiprofen to lower A*β* levels in neuroglioma cells and in AAP transgenic mice showed that some but not all of the NSAIDs tested lowered the A*β* in cells and were able to reduce the A*β* levels in the mice [185]. Neurons that were pretreated with ibuprofen showed decreased production of A*β* upon exposure to the cytokine TNF-*α* as compared to untreated neurons [186]. Another study showed that neurons that were treated with COX-1 inhibitors, such as ibuprofen and acetyl salicylic acid, were more resistant to the effects of A*β* than neurons that were treated by COX-2 inhibitors [187]. This study also showed a decrease in the production of prostaglandin E2 in the neurons by treatment of both COX-1 and COX-2 inhibitors.

NSAIDs may also function by activating the peroxisomal proliferators-activated receptors (PPARs), a group of nuclear hormone receptors that act to negatively inhibit the transcription of proinflammatory genes. For example, PPAR*α* agonists have been shown to inhibit IL-6, TNF-*α*, and COX-2 expression in cell cultures [188]. PPAR*γ* has been shown to inhibit microglial activation and a multitude of proinflammatory agents such as cytokines, NOS, and COX-2 [189].

Unfortunately, clinical trials of NSAIDs in AD patients have not been very fruitful [190]. This was especially disappointing in the case of COX-2 inhibitors. A randomized, double-blind, placebo-controlled trial assessing the effect of the COX-2 inhibitor rofecoxib and the COX-1 and COX-2 inhibitor naproxen versus placebo on AD progression did not slow the cognitive decline of patients with mild-to-moderate AD [191]. Another randomized, double-blind, placebo-controlled trial using the COX-2 inhibitor rofecoxib did not slow the decline of AD [192]. Specific A*β*-lowering NSAIDs may need to be used in future clinical trials to see if they are clinically as effective. One possible hypothesis would be for NSAIDs to help in reducing the incidence of the disease, but NSAIDs would not be as useful once the disease occurs.

### 3.2. Glucocorticoid Steroids

Steroids are considered to be potent anti-inflammatory agents and function by regulating the transcription of assorted inflammatory molecules, inhibiting the production of enzymes which mediate prostaglandin production. Steroids also have an effect by reducing the expression of cytokines and complement proteins that are proinflammatory [179]. It is therefore surprising to find that the epidemiological data for the effect of the use of glucocorticoid steroids in the AD brain show either a very weak benefit in the patient [193] or might even show a possible harmful effect [194]. While glucocorticoids were shown to inhibit A*β* induction of chemokines and cytokines in the CNS [195], a randomized, placebo-controlled trial was conducted to determine whether prednisone treatment slowed the rate of cognitive decline in AD patients. This study showed that there was no difference in cognitive decline between the treated and the control groups [196]. Indeed, total levels of the glucocorticoid cortisol in the CF and serum of AD patients were found to be significantly elevated when compared to nondemented control patients [197, 198], suggesting that increased levels of steroids may be associated with AD.

## 4. Flavonoids: A Natural Strategy

A mean by which the proinflammatory responses may be counteracted, and therefore AD's severity reduced, is through a group of natural plant-derived compounds known as polyphenols; specifically those known as “flavonoids” derived from the green tea plant. Flavonoids are a large family of compounds synthesized by plants that have a common chemical structure [199].

Green tea flavonoids like epigallocatechin gallate (EGCG) appear to promote downregulation of innate immune cell functions. Putative mechanisms of flavonoid action on the innate immune system include direct free radical scavenging [200, 201] as well as a reduction of inflammatory cytokine production of molecules including TNF-*α*, IL-1*β*, and prostaglandin E2 [202]. On line with these findings, activated microglia cocultured with neuroblastoma cells were less neurotoxic in the presence of the flavonoid fisetin, suggesting that some flavonoids may act to inhibit proinflammatory innate immune responses [202, 203].

Some flavonoids, including EGCG, may modulate T-cell response by downregulating innate immune response, by stimulating the cytokines that promote Th1 immunity (e.g., TNF-*α*) and by promoting Th2 cytokines. These effects are thought to be mediated in part through the downregulation of NFkB signaling [204–206]. EGCG inhibits TNF-*α*-induced production of MCP-1 from vascular endothelial cells [207]. Furthermore, EGCG also displays the ability to suppress neuron death mediated by activated microglia [208].

Although flavonoid-rich diets and flavonoid administration prevent cognitive impairment associated with inflammation in animal studies [209–211], retrospective cohort studies are inconsistent in showing an inverse association between dietary flavonoid (e.g., green tea) intake and dementia or neurodegenerative disease risk in humans [212–215]. An epidemiological study of Dutch adults, for example, found that total dietary flavonoid intake was not associated with the risk of developing AD [212, 213]. This relation does not include current smokers whose risk of AD decreased by half for every 12 mg increase in daily flavonoid intake. Elderly French men and women with the lowest flavonoid intakes, on the other hand, had a 50% higher risk of developing dementia over the next 5 years than those with the highest intakes [214]. Thus, future human studies (ideally randomized clinical trials) will be required. These studies should involve supplementation with relatively high doses of specific purified flavanoids to shed light to the apparent inverse risk relationship with AD (and whether this occurs by reducing inflammation) and also to determine if such compounds are therapeutically beneficial.

## 5. Conclusions

Increasing concurrent evidence suggests that inflammation significantly contributes to the pathogenesis of AD. The generation and secretion of proinflammatory mediators may interact at multiple levels with neurodegeneration. Thus, proinflammatory cytokines may not only contribute to neuronal death, but they might also influence classical neurodegenerative pathways such as APP processing and *τ* phosphorylation.

The concomitant release of anti-inflammatory mediators may partly antagonize this action ultimately leading to chronic disease. Future studies need to determine whether the course of AD can be influenced by anti-inflammatory treatment strategies, and clinically novel approaches to analyze early neuroinflammation in the human brain are needed to improve how to monitor and control treatment strategies that are targeting inflammatory mechanisms.

##  Conflict of Interests

Authors declare no competing financial interests.
